# A *Dpagt1* Missense Variant Causes Degenerative Retinopathy without Myasthenic Syndrome in Mice

**DOI:** 10.3390/ijms231912005

**Published:** 2022-10-09

**Authors:** Lillian F. Hyde, Yang Kong, Lihong Zhao, Sriganesh Ramachandra Rao, Jieping Wang, Lisa Stone, Andrew Njaa, Gayle B. Collin, Mark P. Krebs, Bo Chang, Steven J. Fliesler, Patsy M. Nishina, Jürgen K. Naggert

**Affiliations:** 1 The Jackson Laboratory, Bar Harbor, ME 04609, USA; 2 The Graduate School of Biomedical Science and Engineering, University of Maine, Orono, ME 04469, USA; 3 Departments of Ophthalmology and Biochemistry and Neuroscience Graduate Program, Jacobs School of Medicine and Biomedical Sciences, State University of New York at Buffalo, Buffalo, NY 14203, USA; 4 Research Service, VA Western New York Healthcare System, Buffalo, NY 14215, USA

**Keywords:** DPAGT1, congenital disorders of glycosylation, sensitized chemical mutagenesis screen, mouse genetics, inherited retinal disease, ER stress

## Abstract

Congenital disorders of glycosylation (CDG) are a heterogenous group of primarily autosomal recessive mendelian diseases caused by disruptions in the synthesis of lipid-linked oligosaccharides and their transfer to proteins. CDGs usually affect multiple organ systems and vary in presentation, even within families. There is currently no cure, and treatment is aimed at ameliorating symptoms and improving quality of life. Here, we describe a chemically induced mouse mutant, *tvrm76,* with early-onset photoreceptor degeneration. The recessive mutation was mapped to Chromosome 9 and associated with a missense mutation in the *Dpagt1* gene encoding UDP-N-acetyl-D-glucosamine:dolichyl-phosphate N-acetyl-D-glucosaminephosphotransferase (EC 2.7.8.15). The mutation is predicted to cause a substitution of aspartic acid with glycine at residue 166 of DPAGT1. This represents the first viable animal model of a *Dpagt1* mutation and a novel phenotype for a CDG. The increased expression of *Ddit3*, and elevated levels of HSPA5 (BiP) suggest the presence of early-onset endoplasmic reticulum (ER) stress. These changes were associated with the induction of photoreceptor apoptosis in *tvrm76* retinas. Mutations in human *DPAGT1* cause myasthenic syndrome-13 and severe forms of a congenital disorder of glycosylation Type Ij. In contrast, *Dpagt1^tvrm76^* homozygous mice present with congenital photoreceptor degeneration without overt muscle or muscular junction involvement. Our results suggest the possibility of *DPAGT1* mutations in human patients that present primarily with retinitis pigmentosa, with little or no muscle disease. Variants in DPAGT1 should be considered when evaluating cases of non-syndromic retinal degeneration.

## 1. Introduction

Congenital disorders of glycosylation (CDG) are a group of more than 160 rare genetic diseases [1,2,3,4], associated with more than 103 different genes [5], which are caused by defects in glycosylation [1,6]. Currently there are no cures and treatment is focused on reducing symptoms and improving quality of life [7]. In specific cases, dietary supplementation [8] or organ transplantation [9] may be helpful. A critical need in the path toward better treatments is the development of cell and animal models to better understand disease pathogenesis and to help develop drugs [5].

CDG Type 1 are a family of autosomal recessive diseases caused by defects in the synthesis of dolichol lipid-linked oligosaccharides and their transfer to proteins in the endoplasmic reticulum (ER) [10,11,12]. Currently there are 28 genes known to cause the disease (OMIM PS212065) whose presentation is highly variable, even within families that carry the same mutation [12,13,14]. In this study, we primarily focus on the role of a dolichol phosphate N-acetylglucosamine-1-phosphotransferase (DPAGT1) variant in the pathological events leading to retinal degeneration, which has yet to be fully elucidated.

The DPAGT1 gene encodes an integral ER membrane enzyme, which is ubiquitously expressed and catalyzes the first step of *N*-glycosylation [1], the predominant form of glycosylation in eukaryotes which governs protein folding and quality control [15]. Glycosylation is an essential process in growth and development, and the most common post-translational modification of lipids and proteins [16]. In human populations, DPAGT1 missense mutations are associated with limb girdle congenital myasthenic syndrome-13 (CMS13; OMIM 614750) and congenital disorders of glycosylation type Ij (CDG1J; OMIM 608093), both characterized by defects in cognitive and in motor abilities [17,18,19,20]. Retinal abnormalities, as a prominent pathological feature, are occasionally noted in CDG patients [21,22]. DPAGT1 is the primary mechanistic target of tunicamycin, a pharmacological agent which triggers secondary ER stress and the unfolded protein response (UPR) by blocking DPAGT1-dependent protein glycosylation [15,23]. Intravitreal injection of tunicamycin causes retinal degeneration driven by opsin hypoglycosylation [24,25], yet DPAGT1 function in the neural retina has not been thoroughly investigated.

Perhaps not surprisingly, given the critical role of DPAGT1 in glycosylation, null mutations in *Dpagt1* lead to embryonic lethality in mice [26]. However, through the Translational Vision Research Models (TVRM) mutagenesis program [27,28], we identified a viable mouse line, *tvrm76*, which harbors a recessive D166G missense mutation in *Dpagt1*. The *tvrm76* mutants were initially identified by a grainy fundus appearance with an attenuation of retinal vessels by indirect ophthalmoscopy. This clinical phenotype was associated with an early-onset photoreceptor degeneration, as assessed by histology. Biochemical characterization of the mutant revealed that increased ER stress may contribute to the cell death observed in the retina. The *tvrm76* mutant is the first viable *Dpagt1* model, and further study may provide additional insights into the role of glycosylation in retinal health and the mechanism through which *Dpagt1^tvrm76^* mediates its effects on photoreceptor degeneration, serving as a unique model for investigating retinopathies reported in CDG patients.

## 2. Results

### 2.1. Homozygosity of the Dpagt1^tvrm76^ Allele Causes Early Onset Retinal Degeneration

**Figure 1:**
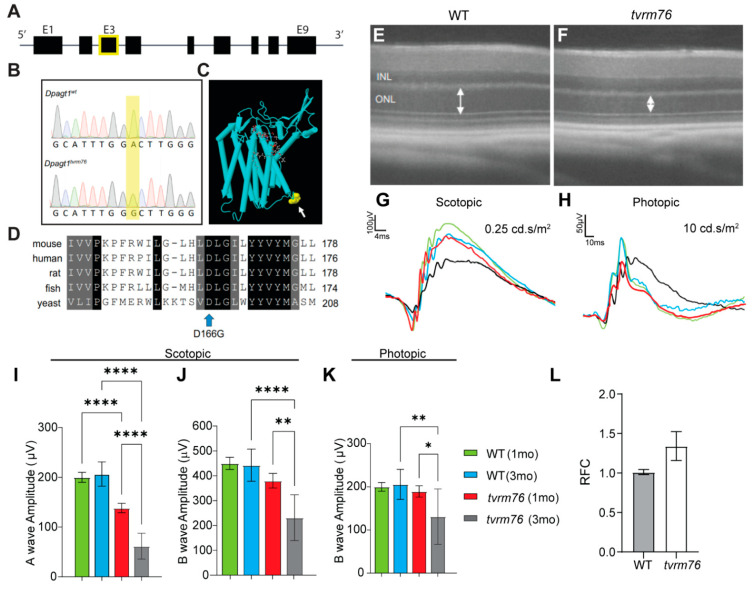
Identification of the *tvrm76* mutation and phenotypic characterization in the retina. (**A**) Genomic structure of *Dpagt1*. Whole exome sequencing revealed a missense mutation in exon 3 of *Dpagt1* (E3, boxed in yellow) (**B**) The mutation results in a transition of adenine to guanine (c.497A > G, shaded in yellow) resulting in an amino acid substitution (aspartic acid (**D**) to glycine (**G**)) at residue 166. (**C**) DPAGT1 protein structure. Yellow arrow indicates location of variant domain. (**D**) Alignment of DPAGT1 amino acid sequences from different species around the *tvrm76* variation. Blue arrow indicates location of D166G missense mutation. (**E**,**F**) A representative OCT image of a *tvrm76* mutant compared to a wild-type (WT) littermate control. Double arrows indicate thickness of outer nuclear layer (ONL). INL, inner nuclear layer. (**G**–**K**) ERG recordings in *tvrm76* mutants at 1 and 3 months of age. (**G**,**I**,**J**) Average scotopic rod response (flash stimuli: 0.25 cd.s/m^2^). (**H**,**K**) Average photopic cone response (flash stimuli: 10 cd.s/m^2^). Results are mean ± SEM. n = 5–9 **** *p* < 0.0001; ** *p* < 0.01; * *p* < 0.05 (Ordinary one-way ANOVA). (**L**) mRNA relative fold change (RFC) of *Dpagt1* by qRT-PCR analysis. Results are mean ± SEM. n = 5 (*p* = 0.0819, Student *t*-test).

Through screening of N-ethyl-N-nitrosourea (ENU) mutagenized C57BL/6J mice by indirect ophthalmoscopy, a strain with pronounced degenerative fundus changes, and progressive pigmentary retinopathy was observed and named *tvrm76*. In order to determine the molecular basis of the *tvrm76* variant, linkage analysis was performed. A recessive locus associated with the retinopathy was identified on Chromosome 9 within an approximately 20 cM region between markers *D9Mit129* and *D9Mit11*. Whole exome sequencing revealed a missense mutation in exon 3 of *Dpagt1,* in which adenine 497 is replaced by guanine (c.497A > G) (Figure 1A,B). The *Dpagt1* mutation co-segregated with the degenerative fundus phenotype in backcross progeny, from the mapping cross, as determined by indirect ophthalmoscopy and by optical coherence tomography (OCT) (Figure 1E,F). The A > G nucleic acid transition is predicted to cause a substitution of an aspartic acid residue by glycine (D166G). By comparison with the 3D structure of human DPAGT1 (UniProt Q9H3H5), the mutated amino acid is located in an interfacial loop between transmembrane ⍺-helical segments 4 and 5 of the DPAGT1 protein, and is conserved among species (Figure 1C,D). Measuring *Dpagt1* expression levels using qPCR showed a slight increase in expression in eyes of mice homozygous for the *tvrm76* mutation relative to wild-type controls, however the difference between groups was not significant (Figure 1L).

In addition to the morphological characterization of the degenerative change, we also assessed retinal function in *tvrm76* mice, using electroretinography (ERG) at one and three months of age. The scotopic a-wave and b-wave responses, associated primarily with rod photoreceptor function, were significantly impaired in one-month-old *tvrm76* homozygotes in comparison to the wild-type littermates, and markedly deteriorated with age (Figure 1G,I,J). Likewise, photopic b-waves were also significantly compromised in *tvrm76* homozygotes at three months of age (Figure 1H,K). The functional losses were concomitant with the observed loss of photoreceptors.

### 2.2. Rod Photoreceptor Specific Loss of DPAGT1 Function Causes Early Onset Retinal Degeneration

**Figure 2:**
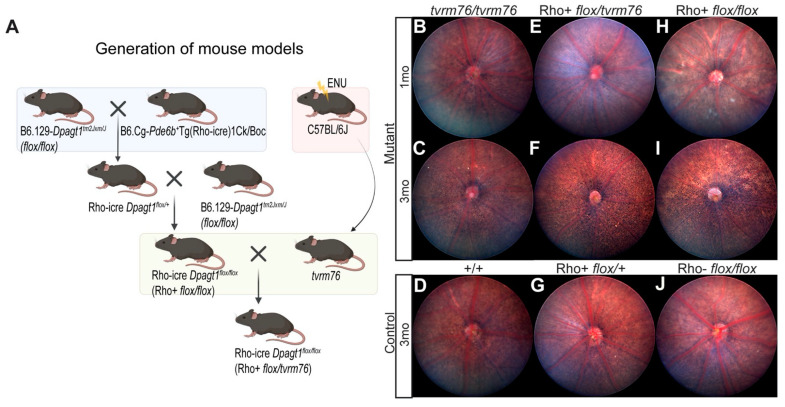
Generation and phenotypic characterization of *tvrm76* and a rod photoreceptor-specific *Dpagt1* conditional knockout by funduscopy. (**A**) Breeding schematic for *tvrm76* and *Dpagt1* conditional knockout models. *tvrm76*, a recessive RP model, was identified through a phenotype-driven screen of putative mutants (putants) generated by chemical mutagenesis (ENU) of C57BL/6J mice and subsequent intercrossing. For localized disruption of *Dpagt1* in photoreceptors, *Dpagt1^tm2Jxm^* was bred with a transgenic mouse expressing rhodopsin-iCre. Subsequently Rho icre (+) *Dpagt1^flox/+^* heterozygous progeny were backcrossed to homozygous *Dpagt-* floxed mice to generate Rho iCre(+)-*Dpagt1^flox/flox^* mice (Rho+ *flox/flox*). For allelic testing, Rho iCre(+)- *Dpagt1^flox/flox^* mice were crossed with a *Dpagt1^tvrm76^* heterozygote to produce Rho iCre(+)-*Dpagt1^flox/tvrm76^*(Rho+ *flox/tvrm76*) compound heterozygotes. (**B**–**J**) Representative funduscopic images of central retina in mutant strains and controls at 1 and 3 months of age. (**B**,**C**) *Dpagt1^tvrm76^* homozygotes, (**D**) wild-type (+/+) littermates. (**E**,**F**) Compound heterozygous (Rho+ *flox/tvrm76*), (**H**,**I**) Rho+ *Dpagt1-*floxed homozygotes and (**G**) Rho+ *flox/+* and (**J**) Rho- *flox/flox* controls.

Understanding the role of *Dpagt1* in vivo is difficult because null mutants are embryonic lethal [26]. To better characterize the effects of the *Dpagt1^tvrm76^* mutation, we created two additional models (Figure 2A). The first, referred to as Rho iCre(+)-*Dpagt1^flox/flox^*, harbors a tissue specific knockout allele of *Dpagt1* in rod photoreceptor cells, and was created by outcrossing a homozygous *Dpagt1* floxed model, B6.129-*Dpagt1^tm2Jxm^*/J, with a transgenic mouse expressing rod photoreceptor-specific Cre recombinase, B6.Cg-*Pde6b*^+^ *Tg(Rho-icre)1Ck*/Boc (PMID15682388) and subsequently backcrossing Rho iCre(+)-*Dpagt1^flox/+^* progeny to homozygous *Dpagt1* floxed mice. The second model, referred to as Rho iCre(+)-*Dpagt1^flox/^*^tvrm76^, bears one conditional null allele of *Dpagt1* and one *Dpagt1^tvrm76^* allele.

To assess the phenotypic changes associated with the *tvrm76* mutation, we performed fundoscopic examinations of *tvrm76* homozygotes at one and three months of age, which showed a progressively grainy appearance, consistent with photoreceptor degeneration [29,30] (Figure 2B,C). Rho iCre(+)-*Dpagt1^flox/tvrm76^* compound heterozygotes (Figure 2E,F) and Rho iCre(+)-*Dpagt1^flox/flox^* homozygotes (Figure 2H,I) displayed a more severe graininess, suggesting advanced degeneration by 3 months of age compared to *Dpagt1^tvrm76^* homozygotes. This was consistent with our expectations that *Dpagt1* is necessary for proper photoreceptor cell viability and function.

### 2.3. A Complementation Test Shows That the Dpagt1^tvrm76^ Mutation Causes the Retinal Degeneration

**Figure 3:**
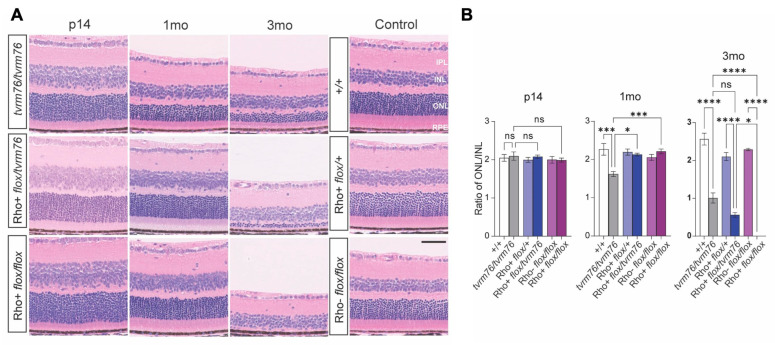
Histological analysis of homozygous *Dpagt1^tvrm76^* and homozygous Rho iCre(+)-*Dpagt1^flox/flox^* models shows progressive photoreceptor degeneration in the mid-periphery of the retina. (**A**) Hematoxylin and eosin (H and E stain) staining of posterior eye sections from *Dpagt1^tvrm76^* homozygotes, compound heterozygotes (Rho+ *flox/tvrm76*) and homozygous Rho iCre(+)-*Dpagt1^flox/flox^* (Rho+ *flox*/*flox*) models at postnatal day 14 (p14), one month (1mo), and three months (3mo) and three month littermate controls. (**B**) Quantification of ONL nuclei degeneration relative to INL. Scale bar = 50 μm. Values represent mean ±SEM; n = 3–6. * *p* < 0.05; *** *p* < 0.001; **** *p* < 0.0001 (one-way ANOVA). IPL: inner plexiform layer; INL: inner nuclear layer; ONL: outer nuclear layer; RPE: retinal pigment epithelium.

To confirm the causal relationship of the *Dpagt1^tvrm76^* allele and the degenerative phenotype observed by funduscopy, we examined retinal histological sections from *Dpagt1^tvrm76^* homozygotes, Rho iCre(+)-*Dpagt1^flox/tvrm76^* compound heterozygotes, Rho iCre(+)-*Dpagt1^flox/flox^* homozygotes, and littermate wild-type controls at postnatal day 14, one month, and three months of age (Figure 3A). At three months of age, the compound heterozygotes had developed pronounced photoreceptor degeneration (Figure 3A), thus confirming that the mutation in *Dpagt1^tvrm76^* leads to retinal degeneration in mice. Photoreceptor degeneration was assessed using the ratio of nuclei from the outer nuclear layer (ONL) to the inner nuclear layer (INL). Measurements were made at three distances from the optic nerve head central (500 µm, Supplementary Figure S1A), mid-peripheral (1000 µm, Figure 3B), and peripheral (1500 µm, Supplementary Figure S1A) to confirm that the effects were consistent across the retina. In the mid-peripheral sections (Figure 3), no differences in the ONL/INL ratio were observed among mutants and their wild-type controls at 14 days of age. At one month, only homozygous *Dpagt1^tvrm76^* mutants showed significant degeneration in contrast to the compound heterozygotes Rho iCre(+)-*Dpagt1^flox/tvrm76^* and homozygous Rho iCre(+)-*Dpagt1^flox/flox^* models, which were similar to wild-type littermates (Figure 3B). The peripheral retina appeared similar among all models at one month (Supplementary Figure S1A). Interestingly, by three months the compound heterozygotes and homozygous Rho iCre(+)-*Dpagt1^flox/flox^* mutants surpassed *Dpagt1^tvrm76^* homozygotes in their degree of photoreceptor degeneration, with the homozygous Rho iCre(+)-*Dpagt1^flox/flox^* mutant having complete ONL loss (Figure 3B). The compound heterozygotes showed a significant thinning of photoreceptors, intermediate between the *Dpagt1^tvrm76^* and Rho iCre(+)-*Dpagt1^flox/flox^* homozygotes in severity. The total loss of photoreceptors observed in the homozygous Rho iCre(+)-*Dpagt1^flox/flox^* model indicates that *Dpagt1* is essential for ONL maintenance, and the slower rate of degeneration in *tvrm76* indicates that the *Dpagt1^tvrm76^* allele with the D166G mutation is hypomorphic and is likely to retain some function.

### 2.4. The Dpagt1^tvrm76^ Variant Is Not Associated with Aberrant Protein Glycosylation at an Early Age

**Figure 4:**
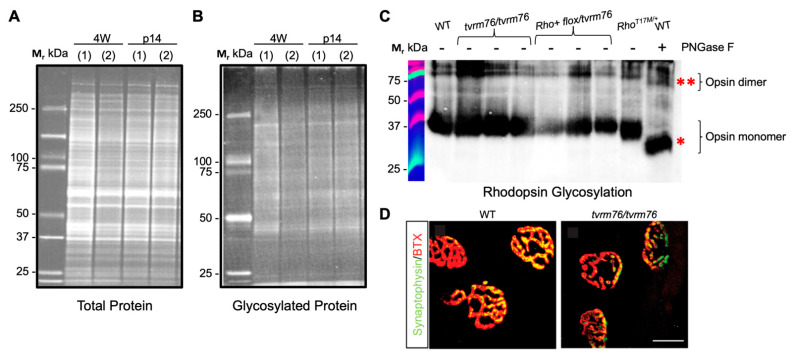
Protein glycosylation analysis in *Dpagt1^tvrm76^* homozygotes. (**A**,**B**) No difference detected in total or glycosylated protein levels observed by SDS-PAGE analyses in *Dpagt1^tvrm76^* homozygotes (2) and their wild-type (+/+) littermates (1) at four and two weeks of age. (**C**) Western blot of rhodopsin from *Dpagt1^tvrm76^ homozygotes* and compound heterozygotes (Rho+ *flox/*tvrm76) shows no glycosylation defect compared to the defect model and positive PNGase control at two weeks of age (n = 3). Asterisks indicate the 1 kDa shift for the opsin monomer (*) and the 2 kDa shift for the opsin dimer (**) after deglycosylation. (**D**) Immunostaining of muscle tissue whole mount (tibialis anterior) dissected from homozygous *Dpagt1^tvrm76^* vs. +/+ against α-Synaptophysin (green) and α-Bungarotoxin (BTX, green). Scale bar = 5 μm.

DPAGT1 carries out the glycan tree transfer (oligosaccharyl transferase activity) in the *N*-linked glycosylation of proteins. Because many retinal proteins are normally glycosylated, their aberrant glycosylation might contribute to the degenerative retinopathy in *Dpagt^tvrm76^* homozygotes. However, qualitative examination of global glycosylation of retinal proteins by western blot analysis at two weeks and four weeks of age failed to show differences in glycosylation between homozygous *Dpagt^tvrm76^* and wild-type mice (Figure 4A,B).

Specific retinal proteins that undergo *N*-glycosylation include opsin. Opsin glycosylation defects lead to autosomal dominant retinitis pigmentosa [31,32,33]. Since it is conceivable that aberrant opsin glycosylation might contribute to the degenerative retinopathy in homozygous *Dpagt1^tvrm76^* mice, we tested the *N-*glycosylation status of opsin. The glycosylation pattern of rhodopsin did not differ between mutants and wild-type at one month of age (Figure 4C). To increase the sensitivity of detecting hypomorphic effects of the *Dpagt^tvrm76^* allele on the *N*-linked glycosylation of rhodopsin, rhodopsin glycosylation was measured at two weeks of age in compound heterozygous (Rho iCre(+)-*Dpagt1^flox/tvrm76^*) mutants. Retinas from Tg(RHO*T17M)5Asl mice [34] served as a positive control, wherein opsin exhibited an electrophoretic mobility shift of ~1 kDa. Additionally, PNGase-F treated wild-type retinal protein lysate (to enzymatically deglycosylate proteins including opsin) served as a true positive control, wherein opsin exhibits a mobility shift of about 2 kDa, in agreement with previous findings [32]. Again, no difference was noted compared to the homozygous *Dpagt1^tvrm76^* and wild-type controls (Figure 4C).

### 2.5. Homozgyous Dpagt1^tvrm76^ Mice Do Not Recapitulate Early Muscular Abnormalities Observed in Patients with DPAGT1 Mutations

Since *DPAGT1* mutations are linked to congenital muscle weakness conditions in the human population, it prompted us to test whether *Dpagt1^tvrm76^* homozygotes had muscle defects at a young age as well. A grip strength survey revealed a similar grip strength between *Dpagt1^tvrm76^* homozygotes and their wild-type littermates at one month of age (Supplementary Figure S2A). Neuromuscular junctions in *Dpagt1^tvrm76^* muscle tissue were also examined. Muscle whole mounts from *Dpagt1^tvrm76^* mutants and controls were stained with anti-synaptophysin, which labels pre-synaptic vesicles, and with α-bungarotoxin (BTX) which binds the acetylcholine receptor (AchR) and localizes to the post-synapse of neuromuscular junctions. Our data showed a similar structural pattern of neuromuscular junctions in *Dpagt1^tvrm76^* homozygotes and wild-type controls (Figure 4D). Transmission electron microscopy of the muscle did not show the tubular aggregates that are often seen in human patients with CMS [18] (Supplementary Figure S2B–E). Within the detection capacity of our assays, homozygous *Dpagt1^tvrm76^* mice at a young age do not appear to recapitulate the early-onset myasthenic conditions associated with aberrant glycosylation in human patients.

### 2.6. Homozygous Dpagt1^tvrm76^ Eyes Show Signs of ER Stress

**Figure 5:**
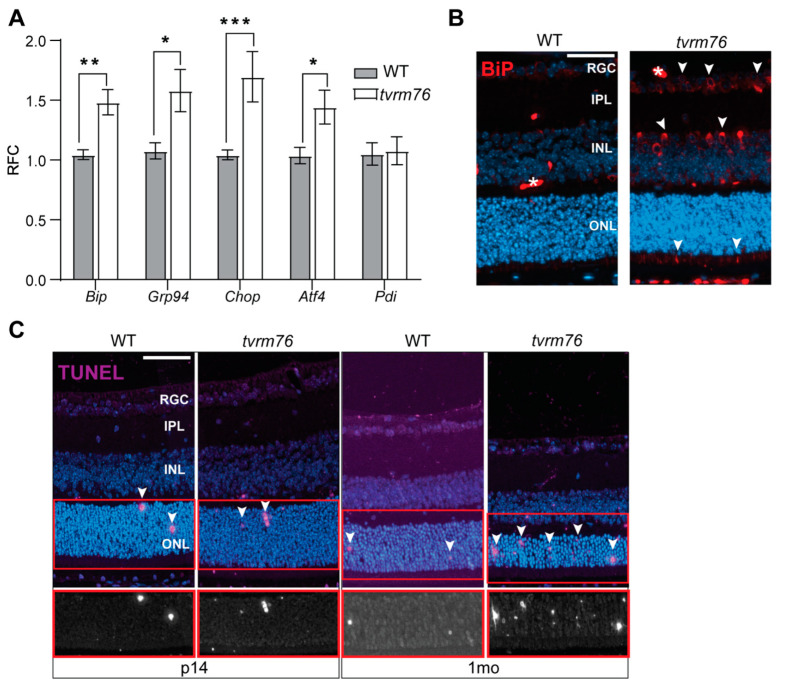
Degenerative retinopathy as a possible consequence of ER-mediated stress response. (**A**) q-RTPCR analysis of ER stress-associated markers in homozygous *Dpagt1^tvrm76^* vs. wild-type. Results mean ± SEM (n = 5). Student *t*-test; * *p* < 0.05; ** *p* < 0.01; *** *p* < 0.001. (**B**) Immunostaining of HSPA5 (BiP) (red) in *Dpagt1^tvrm76^* homozygote vs. wild-type shows increased staining in the inner segments, INL, and RGC layers (white arrowheads). Blood vessel (white *). (**C**) TUNEL staining (purple) of cell apoptosis in the retina at fourteen days (p14) and one month (1mo) of age in *Dpagt1^tvrm76^* homozygotes compared with wild-type. Arrows point to apoptotic cells. Boxed area in lower panel shows TUNEL staining (white) without DAPI. Scale bar (**B**,**C**) = 50 μm.

Since our results did not indicate significant defects in protein post-translational modification associated with the *Dpagt1^tvrm76^* variant, we hypothesized that the activation of endoplasmic reticulum stress could mediate the mutation’s pathogenic effects. Defective glycosylation disrupts the normal post-translational modification of proteins and their proper maturation [35]. This disruption can affect the release of the abnormal proteins from the ER and lead to excessive protein accumulation and ER stress [36]. ER stress in turn can induce cell death [37,38,39]. To test if the mechanism underlying the photoreceptor degeneration in *Dpagt1^tvrm76^* eyes might be attributable to a disturbance of ER homeostasis, a panel of ER-stress-associated molecules, including *Hsp90b1 (Grp94), Hspa5* (*Bip), Ddit3 (Chop), Atf4,* and *Padi2* (*Pdi)* were measured at the transcript level by qRT-PCR at two weeks of age, prior to the onset of photoreceptor degeneration (Figure 5A). Notably, *Ddit3* was significantly upregulated in whole eyes of two-week-old animals. *Hsp90b1* and *Hspa5* also showed increased expression relative to wild-type littermate controls.

To localize ER stress within the retinal layers, immunofluorescent staining of HSPA5 (BiP) was carried out. Increased staining was observed in the inner nuclear layer (INL), retinal ganglion cell layer (RGC), and the photoreceptor inner segments of *Dpagt1^tvrm76^* homozygotes were compared to the wild-type littermate controls (Figure 5B). The ER of photoreceptors is known to be localized in the inner segments, coinciding with our prediction of ER stress. Collectively, our observations revealed elevated transcript levels of some ER stress markers in *tvrm76* mutant eyes that may be indicative of ER stress.

### 2.7. Increased Number of Apoptotic Cells Are Detected in Dpagt1^tvrm76^ Retinas

We next sought to determine the pathological consequences that might arise from ER stress. Considering the significant loss of photoreceptors in homozygous *Dpagt1^tvrm76^* retinas, we examined whether cell-induced apoptosis was detectable by a TUNEL assay. A greater number of apoptotic cells were present in *Dpagt1^tvrm76^* retinas compared to their wild-type counterparts at one month of age, which may underlie the observed loss of retinal cells (Figure 5C). The observation of TUNEL staining supports the initiation of cell apoptosis perhaps due to elevated ER stress, and substantiates retinal degeneration starting at a young age in *Dpagt1^tvrm76^* homozygotes.

## 3. Discussion

The *tvrm76* mutant was identified in an ENU mutagenesis screen, as a strain carrying a recessive mutation causing early-onset retinal degeneration. A missense mutation in *Dpagt1* which is predicted to change aspartate 166 to glycine (D166G), was identified and its causative role in photoreceptor cell loss was confirmed by observing retinal degeneration in compound heterozygous, Rho+ *Dpagt1^flox/tvrm76^*, animals. The D166G mutation lies within the luminal loop between transmembrane segments 4 and 5 of the DPAGT1 protein, which contain residues predicted to interact with the dolichol-phosphate binding site. Furthermore, two severe CDGIj mutations in humans, L168P and Y170C, are localized in close proximity to D166G and retain only 25% of wild-type enzymatic activity [15]. Mutations occurring in these regions often significantly diminish catalytic activity of the molecule, whereas those that are distant from the active sites result in a smaller change in enzymatic activity [15,18,20,40,41]. In a qualitative assessment of glycosylation in homozygous *Dpagt1^tvrm76^* eyes, we saw no noticeable difference in global and rhodopsin glycosylation at two and four weeks of age, respectively, similar to observations made in a model of rod photoreceptor-specific loss of DHDDS, an enzyme preceding DPAGT1 in the glycosylation chain [42]. In the case of *Dpagt1^tvrm76^*, it is, however, most likely that the D166G mutation represents a hypomorphic allele with sufficient activity to maintain near-normal glycosylation levels in the retina.

*Dpagt1^tvrm76^* mutant mice may serve as a novel model for studying degenerative retinopathy due to defects associated with reduced DPAGT1 function. In clinics, many patients bearing mutations in *DPAGT1* manifest multiple neurological symptoms [43,44,45,46]. Previous studies have primarily focused on the muscular presentations, while eye symptoms are rarely addressed [47,48,49,50]. In this study, we found that the *Dpagt1^tvrm76^* model recapitulates the retinal degeneration found in some human patients and enables researchers to better probe the pathogenesis of degenerative changes in the retina caused by *DPAGT1* mutations. *Dpagt1* knockout mice are embryonic lethal [26,51], precluding the study of DPAGT1 in the retina. Therefore, the B6-*Dpagt1^tvrm76^* and the rod photoreceptor-specific B6.129-*Dpagt1^tm2Jxm^*/J models provide a unique opportunity to study DPAGT1 function in this cell type. Future investigations using the rod photoreceptor-specific *Dpagt1* deletion will facilitate the understanding of DPAGT1-dependent protein glycosylation in retinal development and/or maintenance.

Typically, patients harboring *DPAGT1* variants present with limb-girdle weakness [52,53], and retinal degeneration is not noted. In this study, overt myasthenic manifestations were not detected in *Dpagt1^tvrm76^* homozygotes. This is unlikely to be due to the specific affected isoform of DPAGT1, since all annotated splice variants carry the 166G mutant allele. While we only examined the grip strength of *Dpagt1^tvrm76^* homozygotes at one-month of age, at six-months of age, the animals also did not show any apparent muscular weakness compared to the wild-type during normal ambulation. Additional examination at an advanced age would be desirable to assess potential long-term effects of the *Dpagt1^tvrm76^* mutation on muscle function. It should be noted that a large number of patients harboring *DPAGT1* variants were subject to underdiagnosis in clinics as well [45,52,53,54]. Nevertheless, our results demonstrate that *Dpagt1* mutations exist which primarily present with an eye phenotype with minimal muscle involvement. It is possible that the observed phenotype depends on a combination of a specific allele and genetic background modifiers, especially since a variation in disease severity has been observed in human patients [50,52,55].

Proper glycosylation of a large array of proteins is important for their correct folding and stability [16,56,57,58]. Since glycosylation primarily occurs in the ER, accumulation of misfolded proteins there can result in ER stress and the induction of the unfolded protein response (UPR) [59,60,61]. The assessment of ER stress and UPR in human CDG has been carried out using patient-derived fibroblasts [20] and genetically engineered cell lines [62]. These experiments did not show an activation of UPR in fibroblasts carrying *DPAGT1* mutations. However, the mutant cells were more sensitive to the ER stressor tunicamycin than control cells [20]. Concordantly, we observed only a moderate 30–70% increase in the expression levels of ER stress markers and a presence of HSPA5 (BiP)-positive cells in the eyes of homozygous *Dpagt1^tvrm76^* mice. Whether this modest increase in ER stress, unfolded protein response, and DDIT3 (CHOP) activation is solely responsible for the observed apoptosis of photoreceptor cells in our model is currently unconfirmed.

The results presented here demonstrate that *DPAGT1* mutations may exist which predominantly present at an early age as a non-syndromic retinal degeneration. *DPAGT1* should, therefore, be considered as a candidate gene for retinal diseases which map within its vicinity. The *Dpagt1^tvrm76^* model may be useful for pathological studies to better understand how a mutation in *Dpagt1* leads to a retinal degenerative phenotype. In addition, the viability of *Dpagt1^tvrm76^* makes it an attractive model for studying the role of DPAGT1 in other organ systems not examined in this study.

## 4. Materials and Methods

### 4.1. Mice, Mutagenesis, and Husbandry

All of the mice involved in this study were bred and housed in the Research Animal Facility at the Jackson Laboratory, and study protocols used in this project were reviewed and approved by the JAX Institutional Animal Care and Use Committee. Male C57BL/6J (B6/J, 000664) were mutagenized (G_0_) at 10–12 weeks of age by weekly intraperitoneal injections of N-ethylnitrosourea (Sigma-Aldrich, St. Louis, MO, USA) at 70 mg/kg body weight for three consecutive weeks. The G_0_ mice were mated with unmutagenized female B6/J to produce G_1_ progeny, which were subsequently mated to female B6/J to produce a G_2_ population. G_2_ females were backcrossed to their G_1_ sires to produce G_3_ mice, which were screened at 12 weeks for ocular phenotypes by indirect ophthalmoscopy. A G_3_ male mouse was found to have a fundus with grainy appearance. Because the G_3_ mouse did not breed, G_3_ siblings were intercrossed to confirm heritability and establish the line, named *tvrm76* (C57BL/6J-*Dpagt1^tvrm76^*/Pjn), which showed recessive inheritance. From these initial matings, we determined that both homozygous *tvrm76* male and female mice did not breed.

To conditionally knockout *Dpagt1* in rod photoreceptors, mice bearing a *Dpagt1*-floxed allele, B6.129-*Dpagt1^tm2Jxm^*/J (JAX:006887), were crossed to B6.Cg-*Pde6b^+^* Tg(Rho-icre)1Ck/Boc (JAX:015850) and backcrossed to generate mice homozygous for the floxed *Dpagt1* allele and carrying the rod-expressing Rho-icre transgene (Rho iCre(+)-*Dpagt1^flox/flox^*).

### 4.2. Identification of the Molecular Basis of tvrm76

Due to the infertility of homozygous *tvrm76* mutants, progeny-tested heterozygous mice were used in mapping experiments. C57BL/6J-*Dpagt1^tvrm76^*/Pjn heterozygotes were outcrossed to strain DBA/2J and resulting F1s were either backcrossed to C57BL/6J-*Dpagt1^tvrm76^*/Pjn heterozygotes or B6:D2-*Dpagt1^tvrm76^* heterozygotes. In the former instance, only affected mice (grainy fundus) were used for linkage analysis. The chromosomal location of the *tvrm76* locus was identified by genotyping 48 MIT markers across the genome in 24 affected mice from the linkage crosses. To identify the causative mutation, high-quality genomic DNA was prepared from the tails of *tvrm76* homozygotes for the preparation of exome capture libraries which were sequenced on a HiSeq 2000 sequencing system (Illumina, San Diego, CA, USA), as previously described [63]. Whole-exome sequencing of *tvrm76* identified a missense mutation in *Dpagt1*, which was subsequently confirmed by Sanger sequencing.

### 4.3. Phenotyping

The fundus of mouse eyes was examined using a Heine Omega 500 indirect ophthalmoscope with a 60-diopter aspheric lens [64]. Fundus images were captured using a Micron IV camera (Phoenix Research Laboratories, Vancouver, BC, Canada) after topical administration of 1% atropine sulphate for pupil dilation, and Goniovisc hypromellose ophthalmic solution (Sigma-Aldrich, St. Louis, MO, USA) for surface lubrication and the formation of a fluid bridge, as necessary [65]. Non-invasive sectional examination of mouse retinas was performed using a Bioptigen (Leica Microsystems, Deerfield, IL, USA) ultra-high resolution (UHR) Envisu R2210 spectral domain OCT (SDOCT) [65]. Mice eyes were dilated using 1% cyclopentolate (Akorn, Lake Forest, IL, USA) or 1% atropine (Akorn, Lake Forest, IL, USA) and anesthetized with ketamine/xylazine (100 mg/mL ketamine (Covetrus, Portland, ME, USA), 20 mg/mL xylazine (Akorn, Lake Forest, IL, USA), and 0.9% *w*/*v* sodium chloride). The function of the outer retina was assessed by electroretinography (ERG). *Dpagt1^tvrm76^* homozygotes and their wild-type counterparts were examined at 1 and 3 months of age. ERG was recorded and analyzed following the routine protocol described previously [66]. Photoreceptor degeneration was assessed using the ratio of nuclei from the outer nuclear layer (ONL) to the inner nuclear layer (INL). Measurements were made at three distances from the optic nerve head central (500 µm), mid-peripheral (1000 µm), and peripheral (1500 µm) to confirm the effects were consistent across the retina. The measure of mouse grip strength was determined by recording the duration of time a mouse held onto an inverted wire mesh screen. The experiment followed a previously published protocol [67]. In brief, *Dpagt1^tvrm76^* homozygotes and their wild-type control mice were placed in the center of a wire mesh screen, which was subsequently inverted (T_0_). The time when the mouse fell off was recorded (T_f_).

### 4.4. Histology

For gross histology, eyes were enucleated from *Dpagt1^tvrm76^* homozygotes and their sibling wild-type controls immediately after carbon dioxide asphyxiation. Tissues were fixed in acetic acid: methanol: phosphate buffer (1:3:4) prior to paraffin embedding and sectioning at 5 microns. Tissue sections were de-paraffinized and stained with hematoxylin and eosin (H and E staining), as previously described [66], and examined by light microscopy. Images were captured using a NanoZoomer 2.0-HT digital slide scanner (Hammamatsu, Shizuoka, Japan) at 40× magnification.

### 4.5. Electron Microscopy

Gastrocnemius muscle was fixed in a mixture of glutaraldehyde (2.5%) and paraformaldehyde (2%) in a sodium phosphate buffer. The samples were then post-fixed in 1% OsO_4_ and rinsed in a cacodylate buffer. After dehydration, tissue was embedded in Epon and sectioned for TEM cross-sectionally and longitudinally. Ultrastructure was imaged using a transmission electron microscope (Hitachi HT-7700, Tokyo, Japan).

### 4.6. RNA Extraction and Analysis

RNA was extracted from whole eyes of two-week-old *Dpagt1^tvrm76^* and wild-type littermates using TRIzol (Invitrogen, Waltham, MA, USA, Cat #15596026) and the gentleMACS tissue dissociator (Miltenyi Biotec, Bergisch Gladbach, Germany, Cat #130-093-235). The mRNA was extracted using an RNeasy Mini Kit (Qiagen, Venlo, The Netherlands, Cat # 74104) following the protocol provided by the manufacturer. cDNA was synthesized using the SuperScript cDNA synthesis system (Invitrogen, Waltham, MA, USA, Cat #18091200), and q-RTPCR was performed using iTaq Universal SYBR Green Supermix in the CFX96 Touch Real-Time PCR Detection System (Bio-Rad, Hercules, CA, USA, Cat #1725121). The relative fold change was determined using the comparative CT method (ΔΔC_t_) and normalized to the level of ß-actin (*Actb*) mRNA, an internal control calibrator. The primers are presented in Supplementary Table S1.

### 4.7. Immunofluorescence

Enucleated eyes from two-week- and one-month-old *Dpagt1^tvrm76^* mutants and wild-type littermates were fixed overnight in methanol:acetic acid in phosphate-buffered saline (PBS) at a ratio of 3:1:4. Tissues were subsequently embedded and sectioned at four µm for immunohistochemistry. Slides underwent a standard deparaffination and rehydration protocol. Antigen retrieval was performed using a 10 mM citrate buffer in a water bath set at 100 °C for 30 min. Nonspecific binding sites were blocked using 1:50 normal donkey serum (Jackson ImmunoResearch Laboratories, West Grove, PA, USA) and 0.5% Triton-X (Sigma-Aldrich, St. Louis, MO, USA) in PBS. Antibody incubation was performed, as previously described [66]. To prepare muscle specimens for whole-mount staining, tibialis anterior tissue was dissected and flattened immediately before overnight fixation with 4% paraformaldehyde (PFA, Sigma-Aldrich, St. Louis, MO, USA) in PBS at 4 °C. The muscle tissues were co-immunostained and mounted for imaging according to recommended procedures [68,69]. For detecting apoptotic cells, TUNEL assays were carried out by using the In Situ Apoptosis Detection, Alexa Fluor 647 Dye (Invitrogen, Cat #C10619), following the manufacturer’s protocol. Samples were mounted in Vectashield (Vector Laboratories, Burlingame, CA, USA) and imaged using a Zeiss Axio Observer.z1 fluorescence microscope (Carl Zeiss Microscopy, Jena, Germany). The resulting images were processed in Fiji (https://fiji.sc/#, accessed 3 October 2022) [70] using the Stack Focuser Macro. 

### 4.8. Antibodies

Antibodies used for immunofluorescence staining included rabbit anti-GRP78 (1:200, Cell Signaling, Danvers, MA, USA, Cat #C50B12), fluorophore 594-conjugated α-Bungarotoxin (1:1000, Thermo Fisher Scientific, Waltham, MA, USA, Cat #B13423) and rabbit anti-synaptophysin (1:200, Abcam, Cambridge, UK, Cat #ab32127).

### 4.9. Western Blot

To assess global protein glycosylation, the Pro Q Emerald 488 Glycoprotein Stain Kit (Thermo Fisher Scientific, Waltham, MA, USA, Cat #P21875) was used to stain SDS-PAGE gels with Sypro Ruby, based on the manufacturer’s protocol. Immunoblot analysis for opsin glycosylation was carried out as described elsewhere [32]. Briefly, retinas from C57BL6/J, *tvrm76* and Rho iCre(+)-*Dpagt1^flox/flox^* mice were harvested and flash frozen immediately. Retinas were lysed in RIPA buffer (Thermo Fisher Scientific, Waltham, MA, USA, Cat #89900) supplemented with protease inhibitor cocktail (ThermoFisher Scientific, Waltham, MA, USA, Cat #78441) at 1:100 dilution. Protein content in the lysates was estimated using a Pierce™ BCA Protein Assay Kit (ThermoFisher Scientific, Waltham, MA, USA, Cat #23225). Similarly processed RhoT17M/+ retinas served as a positive control for opsin hypo-glycosylation [34] (Tissues were a kind gift from Dr. Marina Gorbatyuk, UAB, Birmingham, AL, USA). PNGase-F N-glycosidase assay (New England Biolabs Inc., Ipswich, MA, USA; Cat #P0704S) was carried out as per the manufacturer’s instructions. About 20µg of C57BL6/J retinal protein lysate was treated with 200 U of PNGase-F in a 1X Glycobuffer 2 (10X buffer provided with the kit) supplemented with 1% NP-40 detergent at 37 °C, overnight. Protein lysates were subjected to Western blot analysis utilizing the following antibody mouse anti-opsin monoclonal antibody (Abcam, Burlingame, CA, USA; Cat #ab5417, 1:2000). Blots were then probed with appropriate host-specific alkaline phosphatase-tagged secondary antibodies (1 h at room temperature). Detection of antibody binding was achieved using chemifluorescent enzyme substrate (GE Healthcare Life Sciences, Marlborough, MA, USA; Cat #45000947) and a ChemiDoc™ MP Imaging System (Bio-Rad Laboratories, Hercules, CA, USA).

### 4.10. Image Analysis and Statistics

Fiji software (https://fiji.sc/#, accessed 3 October 2022) [70] was used for image processing and western blot quantification. Statistical significance was analyzed by two-way ANOVA with post hoc Bonferroni’s multiple comparisons test, or student-*t* test for comparisons of two groups using GraphPad Prism version 8 (GraphPad Software, San Diego, CA, USA). At least three biological replicates were utilized in each group for statistical analysis. Results are shown as mean ± SEM., *p* < 0.05 was reported as statistically significant.

## Data Availability

Relevant data are contained within the article or Supplementary Materials. Additional datasets generated and/or analyzed for the current study are available from the corresponding author.

## Supplementary Materials

The following supporting information can be downloaded at: https://www.mdpi.com/article/10.3390/ijms231912005/s1.
