# Supplementary material for: A Dpagt1 Missense Variant Causes Degenerative Retinopathy without Myasthenic Syndrome in Mice
Source: Int J Mol Sci. 2022 Oct 9;23(19):12005. doi: 10.3390/ijms231912005 (PMC9570038; doi:10.3390/ijms231912005)
Supplement: Supplementary file 1 [file ijms-23-12005-s001.zip › ijms-1866451-supplementary.pdf]

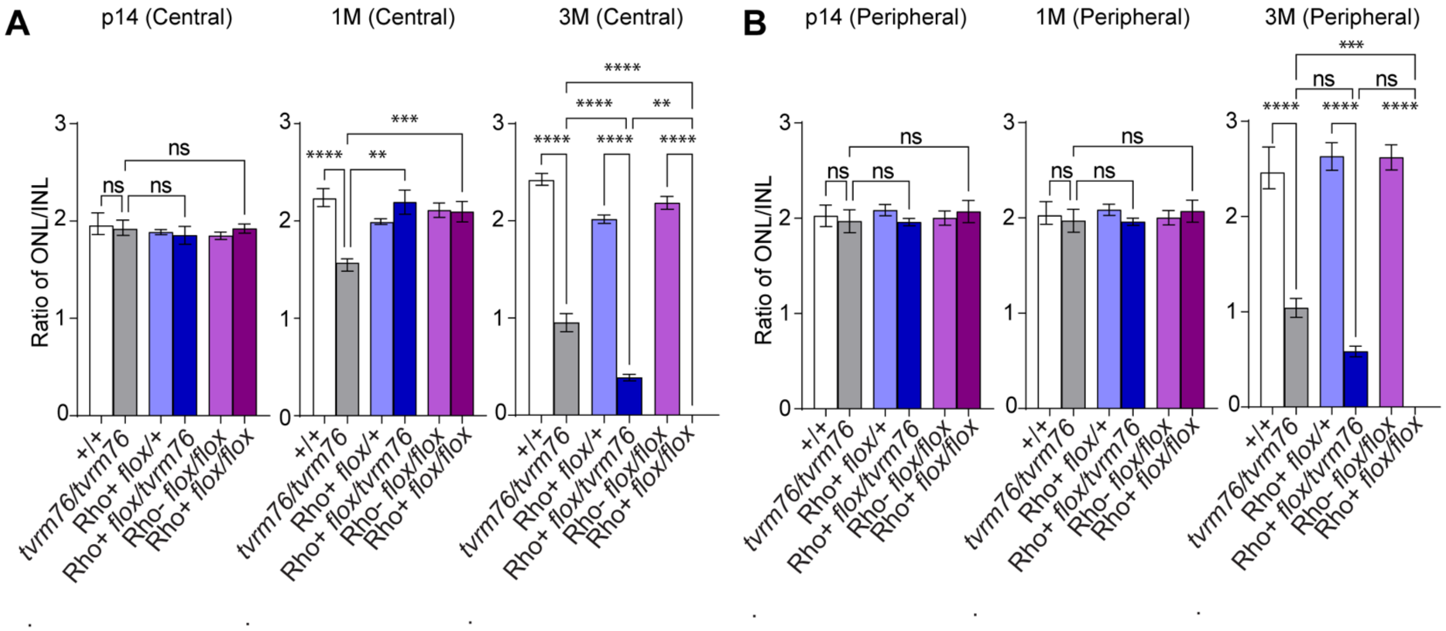

Figure S1. Photoreceptor degeneration in *tvrm76*. (A,B) Quantification of ONL nuclei relative to INL in the central and peripheral retinal regions, respectively. Significant loss of photoreceptors in *tvrm76* mice is observed compared to their wild-type littermates and *Dpagt1* rod photoreceptor knockouts at one month of age in the central retina, while peripheral loss becomes significant at 3 months. Values represent mean ±SEM; n=3-6 **p<0.01;***p<0.001;****p<0.0001 (One-way ANOVA).

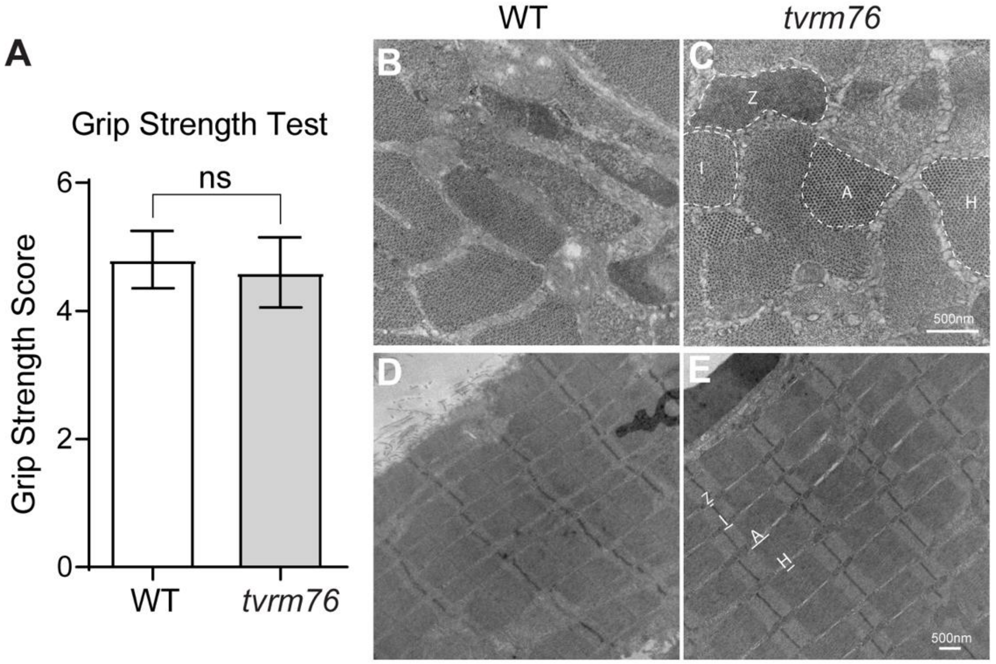

Figure S2. Grip strength and muscular architecture in *tvrm76* and littermate WT controls. **(A)** No significant difference in grip strength of *tvrm76* compared to wildtype controls at 1 month of age. Values represent mean ±SEM; n=5 (p=0.999, Student t-test). **(B-E)** Transmission electron microscopy of gastrocnemius muscle samples in transverse **(B,C)** and longitudinal **(D,E)** sections. No presence of tubular aggregates or abnormal features were observed in *tvrm76* mutants compared to littermate controls at 2 months of age. Z=Z-line, I=I-Band, A=A-Band, H=H-Zone.

Table S1. Primers for qPCR.

**Gene**	**Sequences (5' – 3')**
Actb	F: CCAGTTCGCCATGGATGACGATAT
	R: GTCAGGATACCTCTCTTGCTCTG
Atf4	F: TCGATGCTCTGTTTCGAATG
	R: AGAATGTAAAGGGGGCAACC
Hspa5	F: CCTCTCTGGTGATCAGGATA
	R: CGTGGAGAAGATCTGAGACT
Ddit3	F: CCACCACACCTGAAAGCAGAA
	R: AGGTGAAAGGCAGGGACTCA
Dpagt1	F: GGCCGGTCAGTCACTAGTCA
	R: GGTGTCTCCCACAAACACGC
Hsp90b1	F: GATGGTCTGGCAACATGGAG
	R: CGCCTTGGTGTCTGGTAGAA
Padi2	F: CAAGATCAAGCCCCACCTGAT
	R: AGTTGCCCCAACCAGTACTT
